# Recent Progress in CDK4/6 Inhibitors and PROTACs

**DOI:** 10.3390/molecules28248060

**Published:** 2023-12-13

**Authors:** Hao Wang, Jianfei Ba, Yue Kang, Zeqiao Gong, Tingting Liang, Yahong Zhang, Jianguo Qi, Jianhong Wang

**Affiliations:** Key Laboratory of Natural Medicine and Immuno-Engineering of Henan Province, Henan University Jinming Campus, Kaifeng 475004, China

**Keywords:** cancer, cell cycle, CDK4/6 inhibitors, PROTACs

## Abstract

Cell division in eukaryotes is a highly regulated process that is critical to the life of a cell. Dysregulated cell proliferation, often driven by anomalies in cell Cyclin-dependent kinase (CDK) activation, is a key pathological mechanism in cancer. Recently, selective CDK4/6 inhibitors have shown clinical success, particularly in treating advanced-stage estrogen receptor (ER)-positive and human epidermal growth factor receptor 2 (HER2)-negative breast cancer. This review provides an in-depth analysis of the action mechanism and recent advancements in CDK4/6 inhibitors, categorizing them based on their structural characteristics and origins. Furthermore, it explores proteolysis targeting chimers (PROTACs) targeting CDK4/6. We hope that this review could be of benefit for further research on CDK4/6 inhibitors and PROTACs.

## 1. Introduction

The regulation of eukaryotic cell division involves complex mechanisms, with cyclins and cyclin-dependent kinase (CDK) complexes playing a pivotal role [1]. Since the seminal discovery of cell cycle regulation by Tim Hunt, Paul Nurse, and Leland H. Hartwell—work that garnered a Nobel Prize—20 CDKs have been identified. CDK1-7 and CDK14-18 are primarily associated with cell cycle regulation, while CDK7-13, CDK19, and CDK20 are involved in transcription [2,3,4,5]. Owing to their critical roles in cell cycle progression, cellular transcription, and apoptotic pathways, CDKs have emerged as significant targets in anticancer drug development.

**Figure 1:**
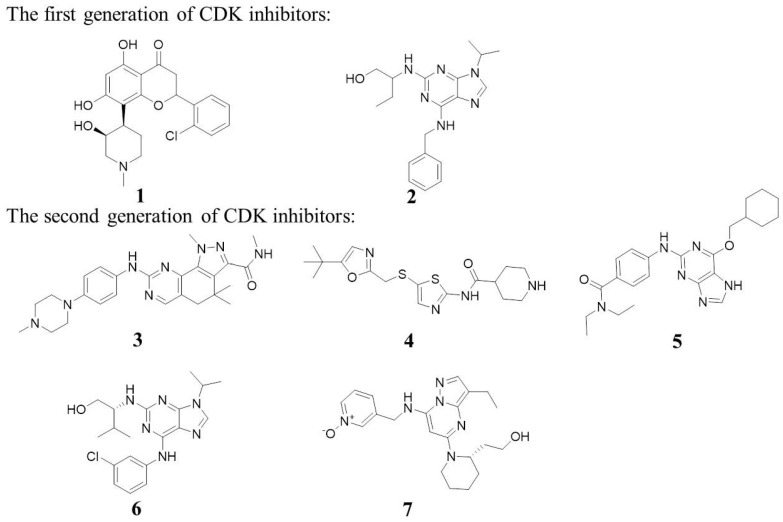
The first and second generations of CDK inhibitors.

Over the past three decades, there has been an intensive search for small molecules that target CDKs. The first-generation compound, Alvocidib (Flavopiridol, Compound **1**, Figure 1), demonstrated inhibition towards multiple CDKs, with IC_50_ values against CDK1, CDK2, CDK4, CDK5, and CDK9 of 30 nM, 170 nM, 100 nM, 170 nM, and 20 nM, respectively. Selliciclib (Roscovitine or CYC202, Compound **2**, Figure 1) shows IC_50_ values against CDK1, CDK2, CDK5, CDK7, and CDK9 of 330 nM, 220 nM, 270 nM, 800 nM, and 230 nM, respectively. However, subsequent preclinical studies revealed limitations like low efficacy and high toxicity. This is due to off-targets and poor pharmacokinetics [6].

The second-generation CDK inhibitors, such as Milciclib (Compound **3**, Figure 1), with IC_50_ values against CDK1, CDK2, CDK4, CDK5, and CDK7 of 53 nM, 45 nM, 140 nM, 170 nM, and 150 nM; SNS-032 (Compound **4**, Figure 1), with IC_50_ values against CDK2, CDK7, and CDK9 of 48 nM, 62 nM, and 4 nM; Nu6140 (Compound **5**, Figure 1), with IC_50_ values against CDK2, CDK4 and CDK5 of 410 nM, 850 nM, and 75 nM; Purvalanol A (Compound **6**, Figure 1), a selective CDK2 inhibitor, with the IC_50_ values of 4 nM; Dinaciclib (Compound **7**, Figure 1), with IC_50_ values against CDK2, CDK5, and CDK9 of 3, 1, 1, and 4 nM, all have their own flaws.

The advent of third-generation CDK inhibitors marked a notable improvement in selectivity, activity, and toxicity. Selective CDK4/6 inhibitors, in particular, have achieved remarkable success in clinical applications, notably in advanced-stage ER-positive breast cancer treatments. Four drugs from this class have been approved by the FDA, with three for cancer and one for myeloprotection [7,8,9,10]. Current research is focused on enhancing the selectivity of CDK inhibitors and addressing drug resistance. There are many excellent reviews on pan-inhibitors, and this review concentrates on selective CDK4/6 inhibitors [11,12,13,14,15].

## 2. The Biological Rationale for Targeting CDK4/6

**Figure 2:**
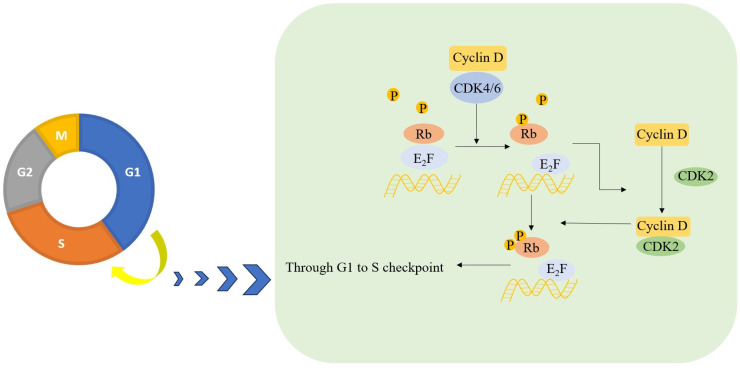
The classical mechanism of CDK4/6 driving the cell cycle.

The classical and best-documented function of CDK4/6 in cell proliferation is that cyclin D1-CDK4/6 phosphorylates the retinoblastoma protein (RB1) and RB-like proteins (RBL1 and RBL2), impacting the G1-S phase transition (Figure 2). The unphosphorylated RB1 interacts with E2F transcription family members, blocking their activity and repressing transcription essential for S phase entry. Phosphorylated RB1, on the other hand, releases E2F, which promotes transcription of cyclin E that associates with CDK2 to further phosphorylate RB1, resulting in the facilitation of the S phase entry. This process is intricately regulated by external signals and mitogenic signaling, with aberrations in the CDK4/6-RB-E2F axis observed in various cancers [16,17].

However, there are some debates surrounding this model. For instance, RB1 is monophosphorylated during the G1 phase and becomes inactivated in the late G1 phase by cyclin E-CDK2, which hyperphosphorylates RB1 on multiple residues. Further, the phosphorylation of RB1 by cyclin D-CDK4/6 is crucial for normal cell-cycle progression, highlighting the need for more research in this area to elucidate the biological function of the CDK4/6-RB axis [18]. 

**Figure 3:**
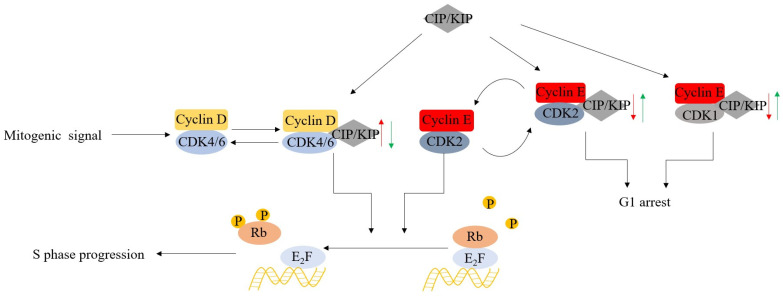
The non-enzymatic function of CDK4/6 on the phosphorylation of RB.

CDK4/6 also influences cell cycle progression through kinase-independent mechanisms. For instance, the Cyclin-dependent kinase inhibitor 1/kinase inhibitory protein (CIP/KIP) protein family, including p21^CIP1^, p27^kIP1^, and p57^kIP2^, binds cyclin E-CDK2 and suppresses its activity (Figure 3). Upregulation of cyclin D and the formation of cyclin D-CDK4/6 complexes, which competitively bind CIP/KIP, leads to redistribution of CIP/KIP, thus activating cyclin E-CDK2 and promoting the G1-S transition.

Beyond facilitating the G1-S transition, Cyclin D-CDK4/6 promotes tumor progression through various pathways. For example, it phosphorylates and stabilizes transcription factor FOXM1, which promotes cell-cycle progression and protects cancer cells from entering senescence [19]. Cyclin D-CDK4 also phosphorylates SMAD3 and inhibits its transcriptional activity, which disables the anti-proliferative ability of growth factor beta (TGF–β) [20]. Cyclin D-CDK4/6 phosphorylates and inactivates tuberous sclerosis complex (TSC2), a negative regulator of rapamycin complex 1 (mTORC1), which subsequently activates mTORC1. CDK6 binds the promoter region of the FMS-like tyrosine kinase 3 (FLT3) gene and the promoter of proviral integration of molony murine leukemia virus 1 (PIM1) pro-oncogenic kinase, stimulating their expression. Treatment of FLT3-mutant leukemic cells with a CDK4/6 inhibitor decreased expression of FLT3 and PIM1, which induced cell cycle arrest and apoptosis [21]. The research on Cyclin D-CDK4/6 promoting tumor progression has also been well reviewed [22]. These diverse roles of CDK4/6 in tumor progression underscore their potential as targets in cancer therapy.

## 3. The Overview of CDK4/6 Sites

All CDKs possess a dual-leaf structure, with the N-terminal comprising β-sheet elements and the C-terminal formed by α-helices. The N-terminal leaf contains G-ring inhibitory components, while the C-terminal leaf is characterized by activating fragments and phosphorylation sites (serine or threonine, referred to as the T-loop). CDK4 is located on chromosome 12q14.1, consisting of a narrow region approximately 3.2 kbp in length. CDK6, mapped to human chromosome 7q21.2, encodes a cytoplasmic protein comprising 326 amino acids and weighing about 37 kDa. The structural and functional similarities between CDK4 and CDK6, with 71% homology in amino acids, facilitate the design of compounds as CDK4/6 inhibitors [23].

**Figure 4:**
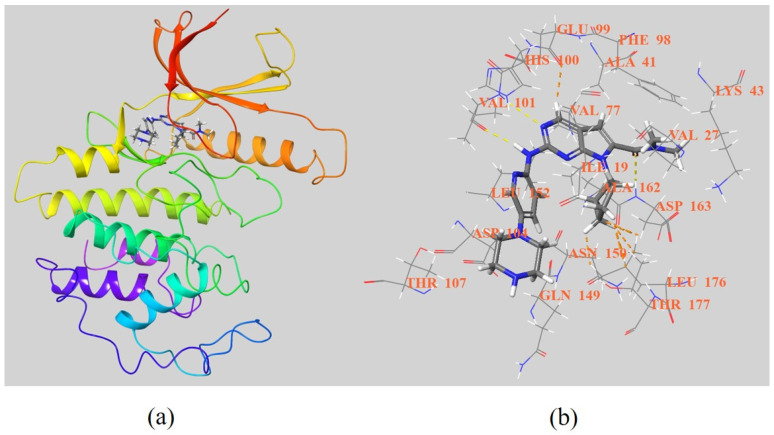
(**a**) The 3D X-ray crystal structure of CDK6 with Ribociclib (PDB ID: 5l2t); (**b**) 3D interaction between CDK6 and Ribociclib. Protein is prepared with discovery studio. Images are prepared with Free Maestro.

The cocrystallization study of Ribociclib and CDK6 (PDB ID: 5l2t) suggests that the 2-aminopyrimidine moiety of Ribociclib forms hydrogen bonds with VAL101, and the pyrrole ring interacts with PHE98, potentially underpinning its inhibitory effect (Figure 4). Additionally, Ribociclib’s piperazine ring, extending outside the protein cavity, seems to play a role in modulating hydrosolubility and selectivity against other CDKs.

## 4. Approved CDK4/6 Inhibitors for Marketing

**Figure 5:**
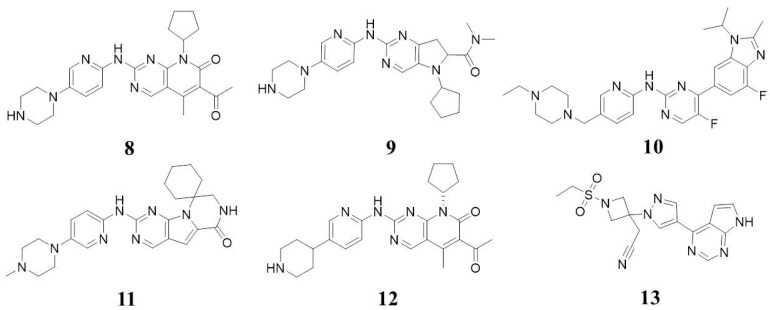
Approved drugs.

The third generation of CDK inhibitors, characterized by enhanced selectivity, reduced side effects, and improved pharmacokinetic properties, has shown significant potential in cancer therapy, particularly in breast cancer. Palbociclib (Ibrance/PD-0332991, Compound **8**, Figure 5), developed by Pfizer, was the first FDA-approved CDK4/6 inhibitor, marking a milestone in the development of these drugs. It exhibits IC_50_ values against CDK4/6 of 11 and 9 nM, respectively [7]. Palbociclib received accelerated approval in the US in February 2015 for first-line treatment of advanced or metastatic ER-positive, HER2-negative breast cancer in postmenopausal women. It has shown efficacy in reducing the proliferation of ER-positive breast cancer cell lines in vitro by blocking Rb phosphorylation, causing G1 phase arrest [24]. Studies also indicate that combined therapy with palbociclib and antiestrogen agents (e.g., letrozole and fulvestrant) leads to a more significant reduction in phosphorylated Rb levels, E2F and FoxM1 levels, and downstream target gene expression. In the xenotransplantation model of ER-positive breast cancer derived from patients, palbociclib plus anti-estrogen letrozole (compared with any drug alone) has a greater inhibitory effect on Rb phosphorylation, downstream signal transduction, and tumor growth [25].

Ribociclib (Compound **9**, Figure 5), developed by Novartis, is another oral, small-molecule inhibitor of CDK4/6, approved in the USA in March 2017 [8]. It displays IC_50_ values against CDK4/6 of 10 nM and 39 nM, respectively. Patient-derived xenograft models of ER-positive breast cancer have demonstrated the enhanced efficacy of ribociclib in combination with antiestrogen agents (letrozole or fulvestrant) and, in some cases, further improvement when combined with a phosphatidylinositol 3-kinase (PI3K) inhibitor [26]. The antitumor effects of ribociclib have also been demonstrated in vitro in leukemia cells.

Abemaciclib (Verzenio/LY2835219, Compound **10**, Figure 5), an oral inhibitor of CDK4/6 developed by Eli Lilly, was approved in the USA on 28 September 2017 [9]. It is indicated in combination with fulvestrant for the treatment of hormone receptor (HR)-positive, human epidermal growth factor receptor 2 (HER2)-negative advanced or metastatic breast cancer, in combination with fulvestrant in women with disease progression following endocrine therapy, and as a monotherapy in adult patients with disease progression following endocrine therapy and prior chemotherapy in the metastatic setting [27,28,29]. It shows IC_50_ values against CDK4 of 2 nM.

Trilaciclib (G1T28, Compound **11**, Figure 5), developed by G1 Therapeutics (formerly G-Zero Therapeutics), is a transient inhibitor of CDK4/6 with IC_50_ values of 1 nM and 4 nM. Trilaciclib induces a transient, reversible G1 cell cycle arrest of proliferating hematopoietic stem and progenitor cells (HSPCs) in bone marrow, protecting them from chemotherapy (myeloprotection) [10]. On 12 February 2021, trilaciclib received its first approval in the USA to decrease the incidence of chemotherapy-induced myelosuppression in adult patients when administered prior to a platinum/etoposide-containing regimen or topotecan-containing regimen for extensive-stage small cell lung cancer (ES-SCLC) [30,31].

Dalpiciclib (SHR6390, Compound **12**, Figure 5), approved by the National Drug Administration (NMPA) on 31 December 2021, shows IC_50_ values against CDK4/6 of 12.4 and 9.9 nM [32]. It exhibits effective antiproliferative activity against various human RB-positive tumor cells, specifically inducing G1 phase arrest and cell aging while reducing the level of Ser780-phosphorylated RB protein.

Birociclib (XZP-3287, Compound **13**, Figure 5), developed by Xuanzhu Biotechnology Co., Ltd., received the NMPA approval in 2022. It is indicated for locally advanced or metastatic adult breast cancer patients with hormone receptor (HR) positive and human epidermal growth factor receptor 2 (Her2) negative who have received two or more endocrine treatments and one chemotherapy in the metastatic setting and disease progression [33].

## 5. Synthesized CDK4/6 Inhibitors 

### 5.1. Structures with 8-Alkyl-2-(arylamino)pyrido [2,3-d]pyrimidin-7(8H)-one

**Figure 6:**
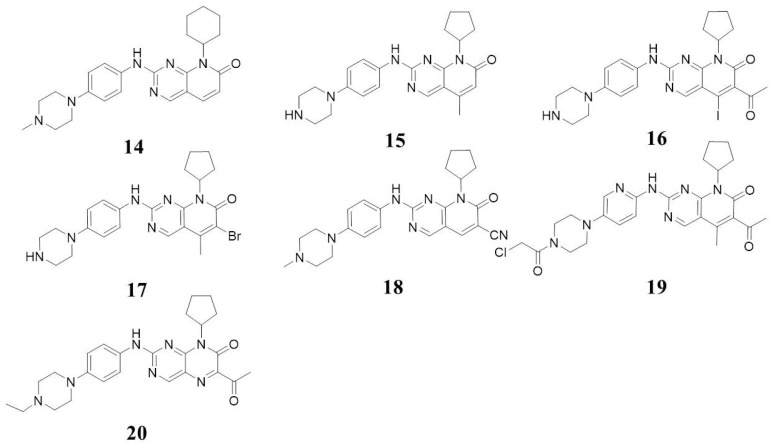
Structures of 8-alkyl-2-(arylamino)pyrido [2,3-*d*]pyrimidin-7(8*H*)-one and the tricycle derivatives.

In 2000, Mark Barvian et al. synthesized a series of pyrimidine-7-ketone compounds. The most potent compound, Compound **14** (Figure 6), exhibited a CDK4 inhibition IC_50_ of 0.004 µM, albeit with moderate selectivity against CDK1/B, CDK2/A, CDK2/E, CDK4/D, and FGFr (IC_50_ values of 0.079, 0.015, 0.020, 0.004, and 0.051 µM, respectively) [34]. Enhancements in selectivity were achieved by introducing a 5-methyl group and replacing cyclohexyl with cyclopentyl on the pyrido [2,3-*d*]pyrimidin-7(8*H*)-one moiety, resulting in Compound **15** (Figure 6), which showed a CDK4/D IC_50_ of 14 nM and >5 µM for CDK1/B, CDK2/A, and CDK2/E [35]. Substitutes in six positions of pyrido [2,3-*d*]pyrimidin-7-ones have been explored. The introduction of bromine, iodine, acetyl, methoxyformyl, and ethoxyformyl all have improved selectivity (Compounds **16** and **17**, Figure 6). Compounds **16** and **17** exhibit IC_50_ values against CDK4 of 4 and 2 nM, respectively [36]. Compound **18** (Figure 6) inhibits CDK4/6 with IC_50_ values of 3.9 and 9.8 nM [37]. In 2021, Huifang Shan et al. designed and synthesized a series of covalent CDK4/6 inhibitors targeting Thr107 amino acids based on palbociclib scaffolds. The optimized Compound **19** (Figure 6) exhibited strong in vitro anticancer activity against CDK4/6. It had inhibitory activity against CDK4/6, with IC_50_ values of 14 ± 1.01 nM and 6.1 ± 0.32 nM [38]. In 2022, Huan He et al. reported pterin-7(8*H*)-one derivatives as CDK4/6 inhibitors, with the most promising Compound L2 (**20**, Figure 6) exhibiting significant inhibitory activity with CDK4 and CDK6 IC_50_ values of 16.7 and 30.5 nM [39]. Based on the above, we may infer that the selectivity of compounds in this class against CDK4/6 has been improved by introducing a small group at the fifth or sixth position on pyrido [2,3-*d*]pyrimidin-7(8*H*)-one moiety, such as methyl, halogen, acetyl, etc.

### 5.2. Structures with a 2-Aminopyrimidin-Containing Tricycle Moiety

**Figure 7:**
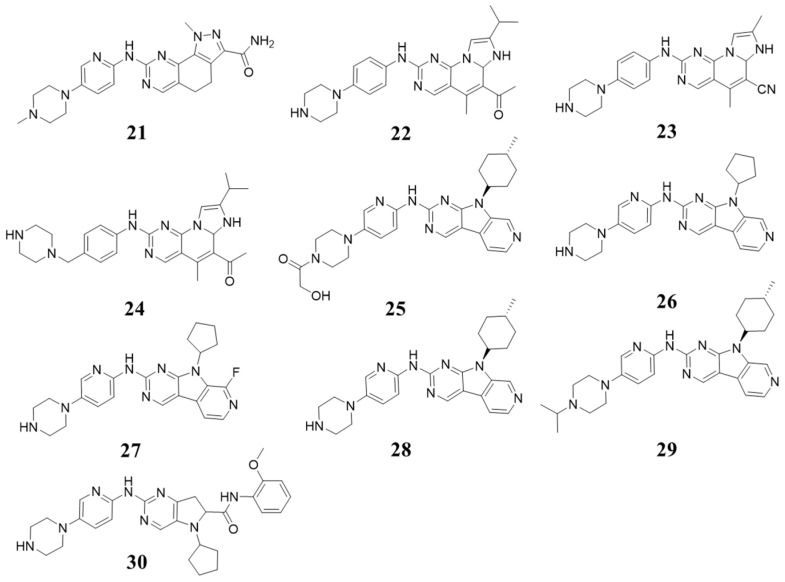
Structures containing pyrido [4′,3′:4,5]pyrrolo [2,3-*d*]pyrimidine and pyrrolidone [2,3-*d*] pyrimidine.

Exploring tricycle moieties based on pyrido [2,3-*d*] pyrimidin-7(8*H*)-one, Hui Zhao et al. in 2018 reported selective 4,5-dihydro-1*H*-pyrazolo [4,3-*h*]quinazoline derivatives. Compound **21** (Figure 7) selectively inhibited CDK4/6 with IC_50_ values of 0.01/0.026 µM [40]. In 2020, Chen Shi et al. synthesized a new series of imidazole [1′,2′:1,6]pyrido [2,3-*d*]pyrimidin derivatives as CDK4/6 inhibitors. Compound **22** (Figure 7) was found to selectively inhibit CDK4/6, with IC_50_ values of 0.8 nM and 2.0 nM. In addition, the bioavailability has improved. Some other tricycle-moiety-containing compounds have been reported. Compound **23** (Figure 7) showed IC_50_ values for CDK4 inhibition of 26.5 nM. Compound **24** (Figure 7) was found to selectively inhibit CDK4/6, with IC_50_ values of 2.2 and 2.5 nM [13,41].

In 2014, Zhihong Li et al. synthesized pyrido [4’,3’:4,5]pyrrolo [2,3-*d*]pyrimidine derivatives, with Compound **25** (Figure 7) inhibiting CDK4 and FLT3 (IC_50_ values of 3 and 1 nM) and exhibiting favorable pharmacokinetics and bioavailability [42]. The structure–activity relationships (SARs) of these compounds were explored, with some (**26**–**29**, Figure 7) showing strong selectivity towards CDK4 (IC_50_ values against CDK4: 2, 3, 7, and 2 nM) [43]. In 2020, Xingpeng Shi et al. designed a series of pyrrolidone [2,3-*d*]pyrimidine with 6-aniline carbonyl substituted derivatives, one of which (Compound **30**, Figure 7) was found to selectively inhibit CDK4/6 with IC_50_ values of 20.5 and 52.3 nM [44]. Based on these compounds, we may infer 9*H*-pyrido [4’,3’:4,5]pyrrolo [2,3-*d*]pyrimidine and 6a,7-dihydroimidazo- [1’,2’:1,6]pyrido [2,3-*d*]pyrimidine is a good replacement for pyrido [2,3-*d*]pyrimidin 7(8*H*)-one without impairment in potency or selectivity.

### 5.3. Structures with 2-(Arylamino)-4-aryl Pyrimidin

**Figure 8:**
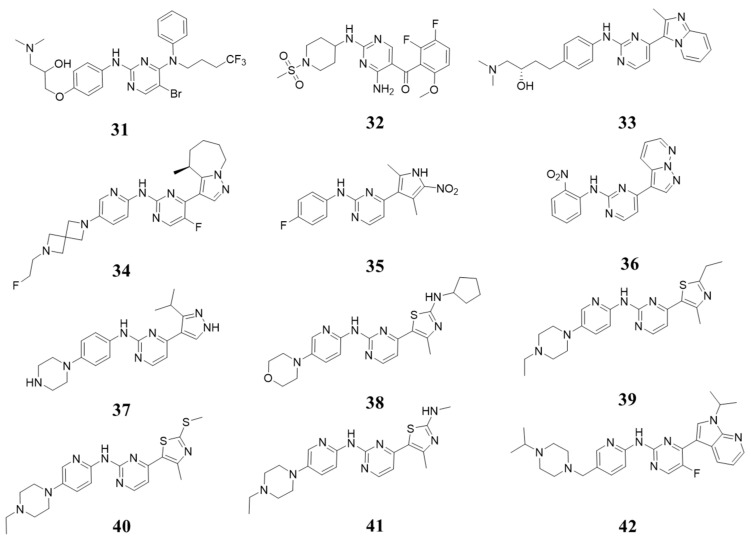
Structures with 2-(arylamino)-4-aryl pyrimidin (a).

Meanwhile, 2-(arylamino)-4-aryl pyrimidin has been explored for the ATP binding site of other kinases; this moiety has been tried for CDK4/6. In 2000, through high-throughput screening, Gloria A. Breault et al. identified a 4,6-bisanilino pyrimidine core structure, with optimized Compound **31** (Figure 8) exhibiting IC_50_ values against CDK2/4 of 200 and 10 nM [45,46]. Xin Jie Chu et al. reported 2,4-diamino-5-ketopyrimidine 6 as a new class of ATP-competitive inhibitors targeting CDK families, with Compound **32** (Figure 8) showing the highest inhibitory activity against CDK4 (IC_50_:1 nM) but also potent against CDK1/2 (Ki = 1/3 nM) [47]. In 2003, Malcolm Anderson et al. discovered Compound **33** (Figure 8), inhibiting CDK4 with an IC_50_ of 0.15 μM [48]. Timothy P. Heffron et al. designed and synthesized a CDK4/6 inhibitor containing an alkaline spirozane structure (**34**, Figure 8), an inhibitor of the cyclin-dependent kinase CDK4/6. It has the strongest inhibitory activity against CDK4/6, with IC_50_ values being less than 0.3 and 1.6 nM [49]. Other aryl groups have been introduced to the four positions of the core structure of 2-(arylamino)-4-aryl pyrimidin, including pyrrole, pyrazol, thiazole, pyrazolo [1,5-*b*]pyridazine, and 1*H*-pyrrolo [2,3-*b*]pyridine. Compound **35** (Figure 8) had inhibitory activity against CDK2/4, with IC_50_ values of 0.03 and 0.12 μM [50]. Compound **36** (Figure 8) had inhibitory activity against CDK2/4, with both IC_50_ values being 0.3 nM [51]. Compound **37** (Figure 8) exhibited an inhibitory effect against CDK1/4, with IC_50_ values of 71 and 5 nM [52]. Compound **38** (Figure 8) was found to selectively inhibit CDK4/6, with IC_50_ values of 4 nM and 30 nM. Furthermore, it has good oral bioavailability [53]. Compounds **39**~**41** (Figure 8) are highly selective CDK4/6 inhibitors. Compound **39** exhibits inhibition activity against CDK4/CDK6, with IC_50_ values of 10 nM and 1.67 µM. Compound **40** exhibits inhibition activity against CDK4/6, with IC_50_ values of 7 and 42 nM [54]. Compound **41** shows IC_50_ values of CDK4/6 at 5 and 50 nM [55]. In 2022, Kai Yuan et al. reported Compound **42**, which had inhibitory activity against CDK4/6 with IC_50_ values of 22 and 10 nM; besides, it had favorable bioavailability and slow clearance with a t1/2 value of more than 24 h in Sprague-Dawley (SD) rats [56].

**Figure 9:**
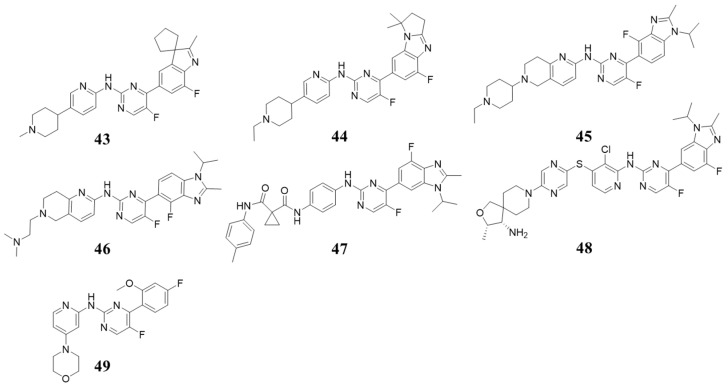
Structures containing 2-(arylamino)-4-aryl pyrimidin (b).

In 2016, Lei Yin et al. reported a series of CDK4/6 inhibitors with notable blood-brain barrier permeability aimed at treating glioblastoma multiforme (GBM). Compound **43** (Figure 9) exhibited good pharmacological characteristics and significant penetration of the blood-brain barrier. It inhibited CDK4/cyclin D1 and CDK6/cyclin D3, with IC_50_ values of 3 nM and 1 nM, respectively [57]. Another highly selective CDK4/6 inhibitor, Compound **44** (Figure 9), exhibited IC_50_ values of 7.4 and 0.9 nM [58]. Compound **45** (Figure 9) showed high selectivity against CDK1/4, with IC_50_ values of 1180 and 1.4 nM [59]. Compound **46** (Figure 9), notable for its high potency and selectivity towards CDK4/6 (IC_50_: 0.710/1.10 nM), also displayed anti-proliferative activity, excellent metabolic properties, and favorable pharmacokinetics [60]. Zhi Huang et al. reported novel inhibitors targeting both CDK4 and VEGFR2, with Compound **47** (Figure 9) inhibiting CDK4 and VEGFR2 at 1 µM by 97% and 95%, respectively [61]. In 2022, Xiaoxing Wu et al. identified a series of SHP2 and CDK4 dual inhibitors, including Compound **48** (Figure 9) with notable SHP2 (IC_50_ = 4.3 nM) and CDK4 (IC_50_ = 18.2 nM) inhibitory activity, for triple-negative breast cancer (TNBC) treatment [62]. In 2023, Junyu Xu et al. reported a series of novel pyrimidin-2-amine compounds for the treatment of glioblastoma (GBM), among which LH20 (**49**, Figure 9) can inhibit the activity of CDK4 and CDK6 and reduce the phosphorylation of Rb [63]. Based on the above, we may infer that groups at four positions on pyrimidine have a great influence on selectivity. Usually, the bulk groups have good selectivity; for example, bicycle and tricycle groups are better than the monocycle moiety.

### 5.4. Structures Containing 5-Amino-3-arylindeno[1,2-c]pyrazol-4(2H)-one 

**Figure 10:**
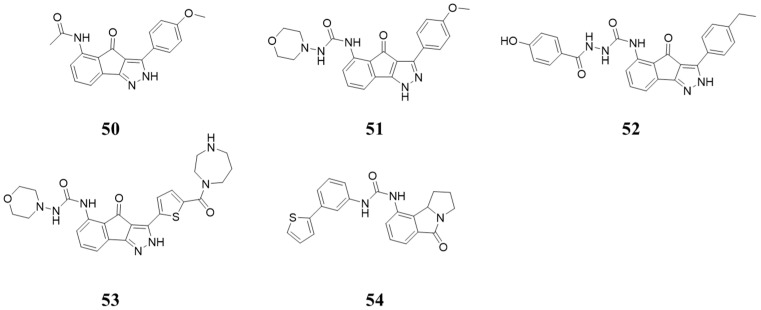
Structures containing 5-amino-3-arylindeno[1,2-*c*]pyrazol-4(2*H*)-one.

In 2001, David A. Nugiel et al. [64] identified Compound **50** (Figure 10) via high-throughput screening and optimization, which showed inhibition against CDK2/4 with IC_50_ values of 0.27 and 0.45 µM. Compound **51** (Figure 10) showed inhibition against CDK2/4, with IC_50_ values of 0.018 and 0.012 µM [65]. The inhibitory activity of Compound **52** (Figure 10) against CDK2/4 with IC_50_ values was 9 and 5 nM [66]. Compound **53** (Figure 10) inhibited CDK2/4, with pIC_50_ values of 8.222 and 8.523 nM [67]. Compound **54** (Figure 10) has an inhibitory effect against CDK4, with IC_50_ values of 0.011 µM. All these compounds have poor selectivity toward CDK2 [68].

### 5.5. Structures Containing 2-Aminothiazole

**Figure 11:**
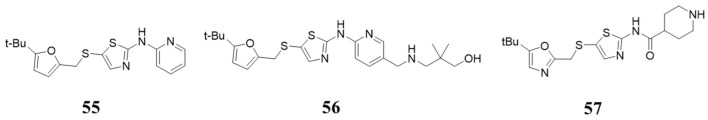
Structures containing 2-aminothiazole.

In 2004, Raj N. Misra et al. synthesized a series of *N*-arylaminothiazole compounds, with Compounds **55** and **56** (Figure 11) exhibiting IC_50_ values against CDK4 of 9 and 26 nM [69]. Compound **57** (Figure 11) had an inhibitory effect against CDK1/2/4 with IC_50_ values of 480, 48, and 925 nM, respectively [70]. Metabolic and pharmacokinetic studies showed that it had a plasma half-life of 5–7 h in three species and exhibited moderately low protein binding in mouse (69%) and human (63%) serum. Oral administration showed 100%, 31%, and 28% bioavailability in mice, rats, and dogs, respectively.

### 5.6. Structures with 8-Alkylamino-quinolin

**Figure 12:**
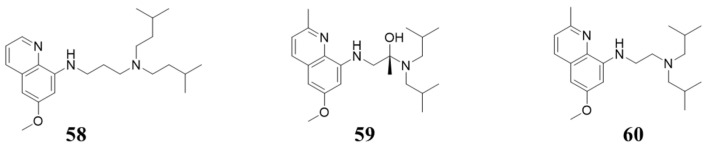
Structures with 8-alkylamino-quinolin.

In 2010, Mark A. Klein et al. identified a series of 8-alkylamino-quinolin compounds based on a pharmacophore established from the peptide p16^INK4a^ (a type of Cip). Compound **58**–**60** (Figure 12) showed inhibition against CDK4, with IC_50_ values of 160, 179, and 128 μM, respectively [71].

### 5.7. Structures Containing (Z)-4-(Aminomethylene)isoquinoline-1,3(2H,4H)-dione

**Figure 13:**
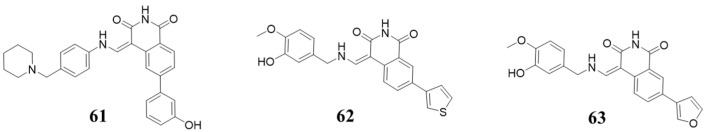
Structures containing (Z)-4-(aminomethylene)isoquinoline-1,3(2*H*,4*H*)-dione.

In 2008, Hwei Ru Tsou et al. designed and synthesized a series of 4-(phenylaminomethylene)isoquinoline-1,3(2*H*,4*H*)-diketone compounds, which selectively inhibit CDK4 over CDK2 and CDK1. The IC_50_ value of Compound **61** (Figure 13) against CDK4 was 27 nM [72]. In 2009, Hwei Ru Tsou et al. designed and synthesized a series of 4-(benzylaminomethylene)isoquinoline-1,3-(2*H*,4*H*)-dione and 4-[(pyridylmethyl) aminomethylene]isoquinoline-1,3-(2*H*, 4*H*)dione derivatives, which effectively and selectively inhibit CDK4 over CDK2 and CDK1. The IC_50_ values of Compounds **62** and **63** (Figure 13) against CDK4 were both 2 nM, while the IC_50_ values against CDK1/2 were 23.3/18.3 and 2.5/1.1 nM [73].

### 5.8. Structures Derived from High-Throughput Screening and Natural Products

**Figure 14:**
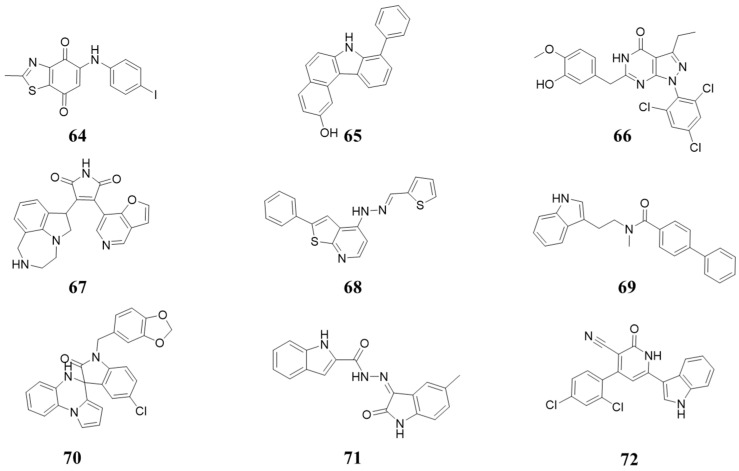
Structures derived from high-throughput screening and natural products.

In 2000, Chung Kyu Ryu et al. reported 5-arylamino-2-methyl-4,7-dioxobenzothia- zole as an inhibitor of cyclin-dependent kinase 4 (CDK4) and a cytotoxic drug. The IC_50_ value of Compound **64** (Figure 14) for CDK4 was 3 µM versus 200 µM for CDK2 [74]. Compound **65** (Figure 14) was identified based on high-throughput screening and exhibited CDK1/2/4 inhibition with IC_50_ values of 95, 97, and 3.6 µM [75]. Compound **66** (Figure 14) was identified based on high-throughput screening and optimization. It exhibited inhibition against CDK1/2/4, with IC_50_ values of 0.2, 0.019, and 0.024 µM [76]. Compound **67** (Figure 14) had an inhibitory effect against CDK4, with a pIC_50_ value of 7.717 µM [77]. Compound **68** (Figure 14) showed an inhibitory effect against CDK4, with IC_50_ values of 0.05 µM [78]. Compound **69** (Figure 14), derived from Fascaplysin, exhibited inhibition activity against CDK2/4, with IC_50_ values of 521 and 6.2 µM [79]. Compound **70** (Figure 14) showed potent cytotoxic activity, and the antitumor mechanism was probed by a docking study with CDK4 [80]. Compound **71** (Figure 14) had good CDK4 inhibition activity, with IC_50_ values of 1.26 µM [81]. In 2023, Mohamed M. Saleh et al. identified Compound **72** (Figure 14), which inhibits CDK4/6 with IC_50_ values against CDK4/6 of 95 nM and 184 nM, respectively. In addition, it inhibited other kinases, such as EGFR, c-Met, and B-Raf [82].

### 5.9. Other Structures

**Figure 15:**
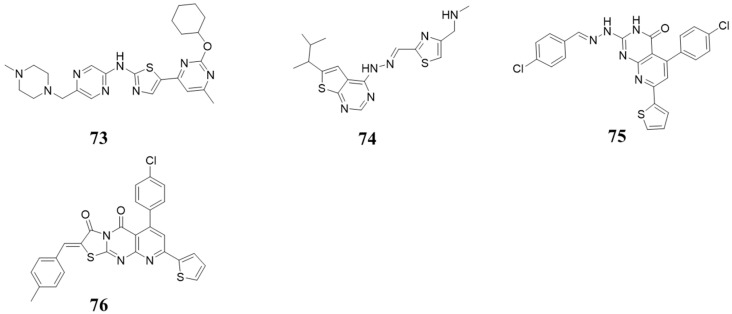
Other structures.

Compound **73** (Figure 15) is a highly selective CDK4/6 inhibitor with IC_50_ values of 9.2 and 7.8 nM, which were found through screening the Merck sample repository and further optimization [83]. In 2015, Takao Horiuchi et al. reported a series of CDK inhibitors, including Compound **74** (Figure 15), which showed CDK2/4 inhibition with IC_50_ values of 880 and 22 nM [84]. Compounds **75** and **76** (Figure 15) exhibited inhibition activity against CDK6, with IC_50_ values of 115.38 nM and 726.25 nM, respectively. Moreover, they increased the levels of bax and p53 and decreased the levels of bcl-2 [85].

## 6. Natural Product Inhibiting CDK4/6

**Figure 16:**
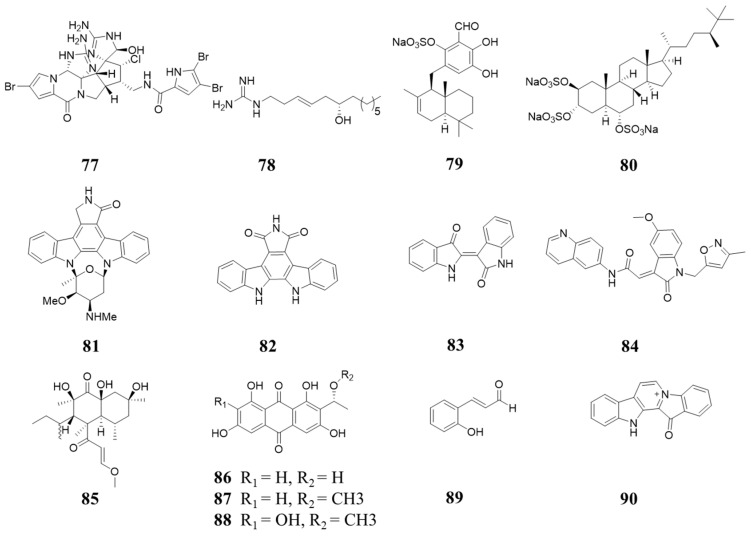
Natural products inhibiting CDK4/6 (a).

In 1997, Jun’ichi et al. reported the isolation and identification of Konbu’acidin A (**77**, Figure 16), which exhibits inhibition activity against CDK4 with an IC_50_ of 20 µg/mL [86]. It is noteworthy that there are two guanidine moieties in the molecule. In 2020, Abdel Nasser B. Singab reported the isolation of Pulchranin A (**78**, Figure 16), which also contains a guanidine moiety. The IC_50_ values of inhibiting CDK1, CDK2, and CDK4 are 9.82, 15.6, and 2.7 µg/mL, respectively [87]. In 2003, Doriano Fabbro et al. reported the isolation of siphonodictyal C (**79**, Figure 16) and halistanol sulfate (**80**, Figure 16) from sponge in Micronesia and inhibition of CDK4/cyclin D1 with 9 and 9.5 µg/mL (IC_50_), respectively. Staurosporie (**81**, Figure 16), an alkaloid from the Streptomyces strain, shows potent CDK4 inhibition activity with an IC_50_ value of 59 nM [88,89]. In 1994, L. M. Ballas reported the isolation of Arcyriaflavin A (**82**, Figure 16) from the marine ascidian (*Eudistoma* sp.), which has a similar aglycone to Staurosporine. It exhibits inhibition of CDK4/cyclin D1 with an IC_50_ value of 140 nM [90]. 

Indirubin (**83**, Figure 16) is the active ingredient of traditional Chinese medicine (i.e., Danggui Longhui Wan), which is used to treat chronic diseases. It interacts with CDK2′s ATP-binding site [91]. Compound **84** (Figure 16), the synthetic analogy of Indirubin, shows potent cytotoxicity against different cancer cell lines. It arrests cells in the G1 phase and is supposed to bind to CDK4 [92]. In 2000, Karsten Schaumann reported four CDK4/cyclin D1 inhibitors produced by the genus Microsphaeropsis isolated from the Mediterranean sponge Aplysina aerophoba, of which one betaenone derivative (**85**, Figure 16) and three 1,3,6,8-tetrahydroxyanthraquinone congeners (**86**–**88**, Figure 16). The IC_50_ values of CDK4/cyclin D1 inhibition activities are 11.5, 43.5, 22.5, and 37.5 µM, respectively [93]. In 2000, Byoung-Mog Kwon et al. found that 2′-hydroxycinnamaldehyde (**89**, Figure 16), isolated from the stem bark of Cinnamonum cassia Blume, showed inhibition activity against CDK4/cyclin D1, with an IC_50_ of 35 µM [94]. The potent and highly specific CDK4/cyclin D1 inhibitor, Fascaplysin (**90**, Figure 16), isolated from the sponge Fascaplysinopsis sp, exhibits CDK4/cyclin D1 inhibition with an IC_50_ of 0.35 µM [95].

**Figure 17:**
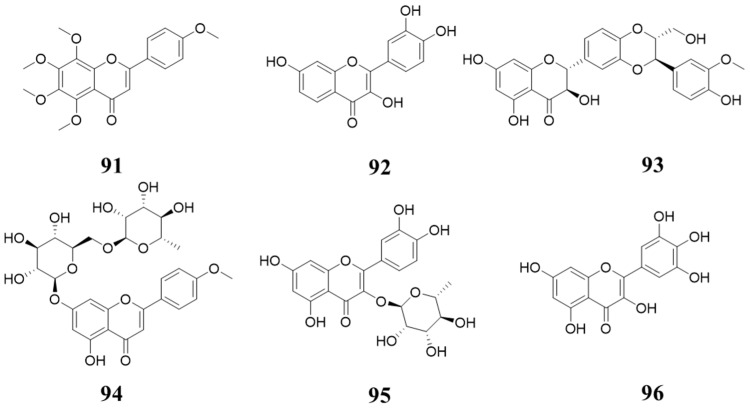
Natural products inhibiting CDK4/6 (b).

Flavonoids are widespread in some traditional Chinese herbs, as well as in some fruit and vegetables. Tangeretin (**91**, Figure 17) is a polymethoxylated flavone that is concentrated in the peel of citrus. It exhibits inhibition activity against CDK2 and CDK4, with IC_50_ values of 26.5 µM and 19.2 µM, respectively [96,97]. In 2005, Jung Han Yoon Park et al. reported that Fisetin (**92**, Figure 17), an ingredient in some fruits and vegetables, inhibits CDK4 activity [98]. Rajesh Agarwal reported that Silibinin (**93**, Figure 17), isolated from milk thistle, shows potent CDK2 and CDK4 inhibition activity [99]. In 2013, Umashankar Vetrivel et al. identified Linarin (**94**, Figure 17) as a potential CDK4 inhibitor via virtual screening [100]. Quercitrin (**95**, Figure 17), isolated from Brownea grandiceps Jacq, also exhibits CDK4 inhibition activity with an IC_50_ of 3.22 µM [101]. In 2020, Ehab M. Mostafa reported that Myricetin (**96**, Figure 17) was isolated from Scorzonera tortuosissima. Boiss inhibits CDK4/cyclin D1 with an IC_50_ value of 3.16 µM [102].

## 7. PROTAC Targeting CDK4/6 and Cyclins

Recently, PROTAC, which degrades CDK4/6, has been considered a strategy to overcome drug resistance. In addition, degradation of CDK4/6 also eliminates other functions of CDK4/6 besides kinase activity, which benefits cancer therapy. 

**Figure 18:**
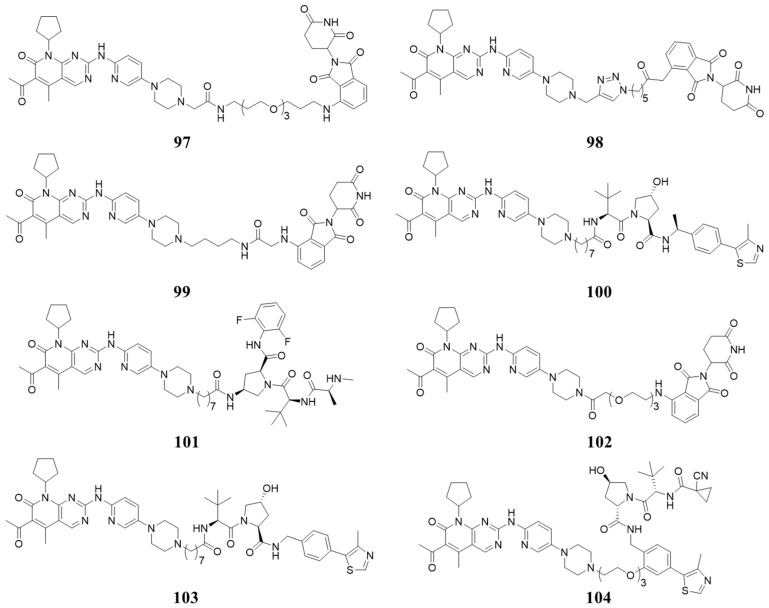
PROTACs targeting CDK4/6.

In 2019, Amarnath Natarajan et al. reported Palbociclib-based PROTAC (**97**, Figure 18) selectively degraded CDK6 while sparing CDK4. It degrades CDK6 as early as 4 h and achieves complete degradation after 8 h at 0.5 µM [103]. Kevin reported Pal-pom (**98**, Figure 18), based on Palbociclib, degraded both CDK4 and CDK6, and degraded CDK4 more efficiently than CDK6 with DC_50_ values of 12.9 and 34.1 nM, respectively (treatment at concentrations of 0.3 µM for 18 h), while the dissociation constants (Kd) for CDK4/6 are 0.9 and 0.2 µM [104]. In 2020, Andrew B. Benowitz reported that Palbociclib-based PROTACs (**99**, Figure 18) degraded CDK4/6 with high binding affinity and degradation potency (pIC_50_ for CDK4/6: 8.5/8.1, pDC_50_ for CDK4/6: 8.0/9.1). Moreover, PROTACs (**100**, **101**, Figure 18) recruiting von Hippel Lindau (VHL) and cellular inhibitor of apoptosis protein (cIAP) ligase have also been prepared, and they showed good degradation potency towards CDK4/6 [105]. Jan Kronke reported systematic research on the structure–activity relationship (SAR) of the linker in the PROTAC targeting CDK4/6. The potent PROTACs (**102**, **103**, Figure 18) were found, with 85%/92.3% and 88%/97.1% degradation of CDK4/6 at 0.1 µM based on different ligases. In addition, the PROTAC (**104**, Figure 18) of CDK6 selective degradation has also been found, with 35%/98.6% degradation of CDK4/6 at 0.1 µM [106].

**Figure 19:**
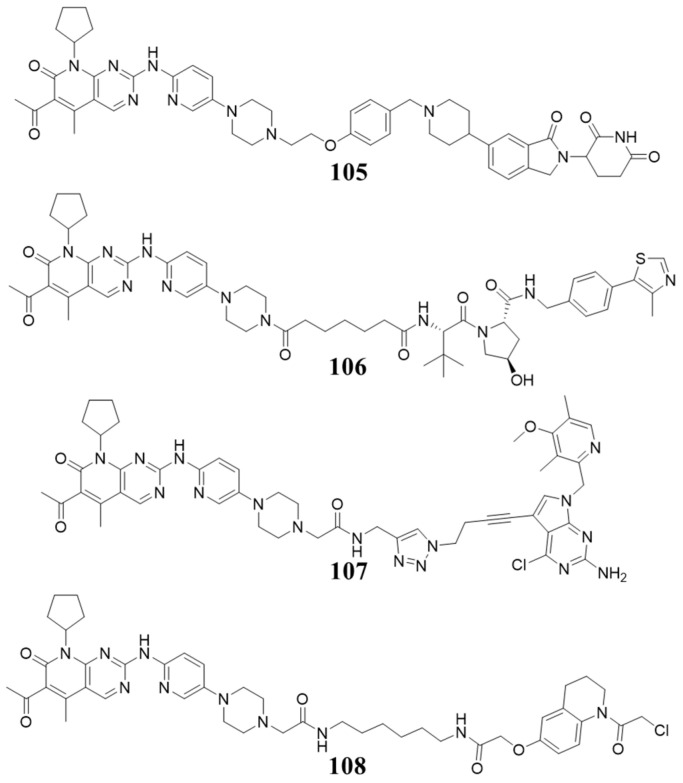
PROTACs targeting CDK4/6 and cyclinD1.

In 2022, Nathanael S. Gray et al. reported a triple degrader (**105**, Figure 19) that consists of palbociclib which could degrade CDK4/6 and Helios [107]. In 2022, Jian Jin reported a palbociclib-based PROTAC (**106**, Figure 19) degraded cyclin D1 via recruiting the CDK4/6-cyclin D1 complex to the VHL ligase, which was considered as an undruggable protein [108]. Recently, the highly CDK4/6 degraders (**107**,**108**, Figure 19) recruiting heat shock protein 90 (HSP90) and DDB1-and CUL4-associated factor 16 (DCAF16) have been reported [109,110].

## 8. CDK4/6 Inhibitors in Clinical Research

In recent years, CDK4/6 inhibitors have been developed rapidly, and some have gradually entered clinical trials. In this section, we list some drugs that have already entered clinical research. 

**Figure 20:**
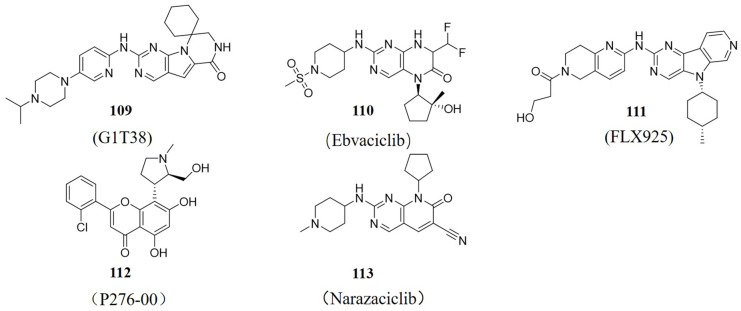
Some structures in clinical research.

G1T38 (Compound **109**, Figure 20, Table 1), developed by G1 Therapeutics ((North Carolina, USA)), is a novel potent CDK4/6 inhibitor with good selectivity and oral bioavailability. At present, it is in a Phase-II-stage clinical study. G1T38 reduced RB phosphorylation, blocked cells in the G1 phase, and inhibited cell proliferation in various CDK4/6-dependent oncogenic cell lines, including breast, melanoma, leukemia, and lymphoma cells. In addition, G1T38 accumulates in mouse xenograft tumors but in plasma, with less neutropenia. All these good pharmacokinetic and pharmacodynamic properties make G1T38 a continuous, daily oral anti-tumor agent [111]. Ebvaciclib (Compound **110**, Figure 20, Table 1), developed by Pfizer (New York, NY, USA), is a CDK2/4/6 inhibitor. In November 2018, the in vitro and in vivo data of Ebvaciclib were first released at the 30th AACR Annual Conference held in Dublin, Ireland. Ebvaciclib has a higher binding affinity for CDK2, 4, 5, and 6, which is 40 times higher than that of CDK1 and CDK9. In March 2018, a Phase-I/II-trial treated patients with HR-positive, HER2-negative breast cancer, metastatic triple-negative breast cancer, or advanced cisplatin-resistant epithelial ovarian cancer/fallopian tube cancer [112].

MM-D37K (Table 1) is a synthetic peptide composed of p16^INK4a^ (a specific inhibitor of cyclin D-CDK4 and CDK6) and cell penetrating peptide (CPP)-Antp (Penetratin). It is a non-ATP-competitive CDK4/6 inhibitor that is in Phase-II-stage clinical research. The merit of MM-D37K over the existing ATP-competitive CDK inhibitors will be explored, benefiting the development of next-generation CDK inhibitors [113].

BPI-16350 (Table 1), developed by Beida Pharmaceutical (Hangzhou, China), is in a Phase-III-stage clinical study. It is applied to treat locally advanced, recurrent, or metastatic breast cancer that has progressed HR+/HER2−, combined with Fluvastatin [114]. RGT-419B (Table 1), developed by Shanghai Qilu Ruige Pharmaceutical Research and Development Co., Ltd. (Shanghai, China), is a potent CDK2/4/6 inhibitor that is in Phase I clinical research. The informed use of RGT-419B is for the unmet medical needs of patients with refractory or recurrent disease after previous treatment and patients with advanced/metastatic breast cancer to improve the survival rate and quality of life [115]. FCN-437c (Table 1) is developed by Fuchuang Pharmaceuticals (Chongqing, China), a subsidiary of Fosun Pharmaceuticals. On 23 January 2019, a Phase I clinical trial was conducted in the United States. In September 2020, the Phase II clinical study was conducted in China for patients with ER+/HER2− advanced breast cancer (excluding Hong Kong, Macao, and Taiwan) [116]. TY-302 (Table 1), developed by Zhengzhou Taiji Hongnuo Pharmaceutical Co., Ltd. (Zhengzhou, China), is a potent and highly selective oral CDK4/6 inhibitor. It was in Phase I clinical trials in December 2019 [117]. TQB3616 (Table 1), developed by Zhengda Tianqing Pharmaceutical (Lianyungang, China), is in Phase III clinical study. It is applied to patients with breast cancer and lung cancer, including HR+/HER2− late/metastatic breast cancer and ER+/HER2+ late/metastatic breast cancer [118]. BEBT-209 (Table 1), developed by Guangzhou Beibeite Pharmaceutical Co., Ltd. (Guangzhou, China), is in Phase II clinical study for patients with advanced breast cancer. Unlike the already-marketed CDK4/6 anti-tumor inhibitors, BEBT-209 improves the selectivity of CDK4 over CDK6, which is expected to reduce the hematological and immunosuppressive toxicity caused by CDK6 activity inhibition [119]. FLX925 (Compound **111**, Figure 20, Table 1), developed by Amgen (FLX BIO) (California, USA), is in Phase I clinical study. FLX925 selectively acts on FLT3 and CDK4/6, and its current indication is acute myeloid leukemia (AML) [120]. P-276-00 (Compound **112**, Figure 20, Table 1), developed by Piramal (Mumbai, India), is in phase II clinical studies for advanced refractory neoplasms and multiple myeloma. It also has inhibitory activity against TNF-α. It also has anti-inflammatory activity, and its first indication for application is for the treatment of mucositis caused by severe radiation in patients with head and neck cancer [121]. SPH4366 (Table 1), developed by Shanghai Pharmaceutical Group (Shanghai, China), is in Phase II/III clinical study. It is used for advanced solid tumor, local, or metastatic breast cancer [122].

ETH-155008 (Table 1), developed by Shengke Pharmaceutical (Suzhou, China), is in Phase I clinical study. It is a PIM3 and CDK4/6 dual-target inhibitor. ETH-155008 further blocks tumor cells in the G1 phase, to some extent avoiding the development of CDK4/6 inhibitor resistance via a synergistic effect [123]. Narazaciclib (ON123300) (Compound **113**, Figure 20, Table 1), developed by Onconova Therapeutics Inc. (Newtown, CT, USA), is in Phase I/II clinical study. It has multi-kinase inhibition activity, including CDK4 (IC_50_ = 3.9 nM), CDK6 (IC_50_ = 9.82 nM), Ark5 (IC_50_ = 5 nM), PDGFR β (IC_50_ = 26 nM), FGFR1 (IC_50_ = 26 nM), RET (IC_50_ = 9.2 nM), and FYN (IC_50_ = 11 nM), with good blood-brain barrier penetration. Narazaciclib inhibits Akt phosphorylation and activates Erk in brain tumors [124]. CGT-1967 (Table 1), developed by Suzhou Shengshi Taike (Suzhou, China), is in Phase I clinical study in China. The informed use is for the treatment of patients with acute myeloid leukemia (AML) [125].

Auceliciclib (Table 1), developed by Ancentra Therapeutics Pty Ltd. (Adelaide, Australia), is currently in phase I/II clinical study [126]. XH-30002 (Table 1), developed by Shanghai Xunhe Pharmaceutical Technology Co., Ltd. (Shanghai, China), is in Phase I clinical trial. Its intended use is to treat advanced solid tumors, including colorectal cancer, breast cancer, ovarian cancer, etc. [127]. HS-10342 (Table 1), developed for Hansen Pharmaceutical (Shanghai, China), is in a Phase I clinical trial. Its intended use is for the treatment of patients with advanced breast cancer with ER+/HER2− [128]. QHRD110 (Table 1), developed by Changzhou Qianhong Biochemical Pharmaceutical (Changzhou, China), is in phase I clinical trials [129]. NUV-422 (Table 1) is a novel inhibitor of the cyclin-dependent kinase CDK2/4/6, developed by Nuvation Bio. (New York, NY, USA). It is a Phase II clinical study for patients with HR+/HER2− advanced breast cancer [130].

## 9. Summary and Prospect

Recently, selective CDK4/6 inhibitors have shown clinical success, particularly in treating advanced-stage estrogen receptor ER+/HER2− breast cancer. Herein, we mainly review the mechanism of action and the progress of CDK4/6 inhibitors. These compounds have been categorized based on molecule similarity and origin. In addition, the proteolysis targeting chimers (PROTACs) targeting CDK4/6 have been reviewed.

However, in the clinic, some patients develop primary or acquired drug resistance. For example, about 20% of breast cancer patients receiving CDK4/6 inhibitor treatment have no response to treatment [131]. These patients already have genetic mutations in their tumor cells, allowing them to avoid the effects of CDK4/6 inhibitors and continue to proliferate in the presence of drugs. Thus far, many primary resistance mechanisms have been identified, all of which seem to involve activation of the cyclin D-CDK4/6-Rb pathway [132].

In addition, the activation of cyclin D-CDK4/6-Rb, activation of other proliferation pathways, changes in the tumor microenvironment, and regulation of tumor metabolism may also lead to the emergence of acquired drug resistance [132]. Within 2 years of starting treatment with CDK4/6 inhibitors in the PALOMA-2 study, over 30% of enrolled patients developed resistance to palbociclib [133]. Furthermore, 40 months later, over 70% of patients in the combination of palbociclib and letrozole group in this study experienced tumor progression during treatment. As prolonged exposure to CDK4/6 inhibitors continues, more and more patients have developed drug resistance; ultimately, all patients receiving CDK4/6 inhibitor treatment will develop acquired resistance [132].

The coming research may involve the following aspects: First, the more potent and selective inhibitors that overcome drug resistance according to the mechanism of drug resistance. Secondly, the dual or multiple targeting inhibitors may have been researched for their synergetic effects or myeloprotection. Finally, the PROTACs may have been tried for anti-resistance based on the action mechanism of PROTAC and the non-enzymatic function of CDK4/6, which benefits cancer therapy.

## Figures and Tables

**Table 1:** Drugs in clinical research.

Drug	R&D Company	Stage of Development	Reference
Lerociciclib (G1T38)	G1 Therapeutics (North Carolina, NC, USA)	Phase II	[111]
Ebvaciclib (PF-06873600)	PFIZER (New York, NY, USA)	Phase I/II	[112]
MM-D37K	MetaMax Ltd. (Moscow, Russia)	Phase I/II	[113]
BPI-16350	Beida Pharmaceutical Company (Hangzhou, China)	Phase III	[114]
RGT-419B	Shanghai Qilu Ruige Pharmaceutical R&D Co., Ltd. (Shanghai, Chian)	Phase I	[115]
FCN-437c	Fuchuang Pharmaceutical Company (Chongqing, China)	Phase I	[116]
TY-302	Zhengzhou Taiji Hongnuo Pharmaceutical Co., Ltd. (Zhengzhou, China)	Phase I	[117]
TQB3616	Zhengda Tianqing Pharmaceutical Co., Ltd. (Lianyungang, China)	Phase III	[118]
BEBT-209	Guangzhou Beibeite Pharmaceutical Co., Ltd. (Guangzhou, China)	Phase II	[119]
AMG925(FLX925)	Amgen (FLX BIO) (San Fernando, CA, USA)	Phase I	[120]
P-276-00	Piramal (Mumbai, India)	Phase II	[121]
SPH4366	Shanghai Pharmaceutical Group (Shanghai, China)	Phase II/III	[122]
ETH-155008	Shengke Pharmaceutical (Suzhou, China)	Phase I	[123]
Narazaciclib (ON123300)	Onconova Therapeutics Inc. (Newtown, PA, USA)	Phase I/II	[124]
CGT-1967	Suzhou Shengshi Taike Company (Suzhou, China)	Phase I	[125]
Auceliciclib	Aucentra Therapeutics (Adelaide, Australia)	Phase I/II	[126]
XH-30002	Shanghai Xunhe Pharmaceutical Technology Co., Ltd. (Shanghai, China)	Phase I	[127]
HS-10342	Hansen Company (Shanghai, China)	Phase I	[128]
QHRD110	Changzhou Qianhong Biochemical Pharmaceutical Co., Ltd. (Changzhou, China)	Phase I	[129]
NUV-422	Nuvation Bio. (New York, NY, USA)	Phase II	[130]
